# D-Cateslytin: a new antifungal agent for the treatment of oral *Candida albicans* associated infections

**DOI:** 10.1038/s41598-018-27417-x

**Published:** 2018-06-18

**Authors:** Pauline Dartevelle, Claire Ehlinger, Abdurraouf Zaet, Christian Boehler, Morgane Rabineau, Benoit Westermann, Jean-Marc Strub, Sarah Cianferani, Youssef Haïkel, Marie-Hélène Metz-Boutigue, Céline Marban

**Affiliations:** 1 INSERM UMR 1121, Biomatériaux et Bioingénierie, Université de Strasbourg, 11 rue Humann, 67085 Strasbourg, France; 2 Faculté de Chirurgie Dentaire, Université de Strasbourg, 3 rue Sainte Elisabeth, 67000 Strasbourg, France; 3 Fédération de Médecine Translationnelle, Université de Strasbourg, Strasbourg, France; 4 Laboratoire de spectrométrie de masse bioorganique, Université de Strasbourg, CNRS UMR 7178 Strasbourg, France

## Abstract

The excessive use of antifungal agents, compounded by the shortage of new drugs being introduced into the market, is causing the accumulation of multi-resistance phenotypes in many fungal strains. Consequently, new alternative molecules to conventional antifungal agents are urgently needed to prevent the emergence of fungal resistance. In this context, Cateslytin (Ctl), a natural peptide derived from the processing of Chromogranin A, has already been described as an effective antimicrobial agent against several pathogens including *Candida albicans*. In the present study, we compared the antimicrobial activity of two conformations of Ctl, L-Ctl and D-Ctl against *Candida albicans*. Our results show that both D-Ctl and L-Ctl were potent and safe antifungal agents. However, in contrast to L-Ctl, D-Ctl was not degraded by proteases secreted by *Candida albicans* and was also stable in saliva. Using video microscopy, we also demonstrated that D-Ctl can rapidly enter *C. albicans*, but is unable to spread within a yeast colony unless from a mother cell to a daughter cell during cellular division. Besides, we revealed that the antifungal activity of D-Ctl could be synergized by voriconazole, an antifungal of reference in the treatment of *Candida albicans* related infections. In conclusion, D-Ctl can be considered as an effective, safe and stable antifungal and could be used alone or in a combination therapy with voriconazole to treat *Candida albicans* related diseases including oral candidosis.

## Introduction

The excessive use of antifungal agents, compounded by the shortage of new drugs being introduced into the market, is causing the accumulation of multi-resistance phenotypes in many fungal strains^1^. Infections caused by these resistant microorganisms often no longer respond to conventional treatment, therefore lengthening the duration of illness related to the infection. Moreover, the widespread use of antifungal agents in clinics and hospitals promotes the development and spread of antifungal-resistant strains and thus the occurrence of nosocomial infections. The development of new alternative molecules to conventional antifungal agents therefore constitutes a major public health issue.

The human oral microbiome is a complex ecosystem made up of several hundred species of microorganisms^2,3^. Especially, this commensal flora plays a key role in maintaining oral homeostasis. However, the disturbance of this balance may cause serious infections including oral candidosis, one of the most prevalent opportunistic fungal infections affecting the oral cavity. Usually, oral candidosis only affects mucosal linings in an inflammatory process, but the rare systemic manifestations may have a fatal course^4,5^. Actually, over time, the microbial plaque forms on the tooth surface and on the oral mucosa. As a matter of fact, a local environment less exposed to the cleansing action of saliva, favours an important release of virulence factors by the pathogens of the plaque, and especially the most commonly isolated microorganism, *Candida albicans*, leading to inflammation of the mucosa and the onset of oral candidosis^6^. In addition, various factors, including age, diabetes, or medical treatments such as chemotherapy and corticosteroids trigger a decrease in the amount of saliva secreted in the oral cavity and are considered as predisposing factors for oral candidosis.

Antifungal medications are often prescribed to treat oral candidosis. In fact, voriconazole constitutes an antifungal of reference to treat *Candida albicans* related infections. However, the prevalence of *Candida* species that are resistant to antifungal agents is increasing, making treatment options a concern^7–10^. Consequently, new alternative molecules to conventional antifungal agents used in dental practice are urgently needed to prevent the emergence of fungal resistance.

Naturally occurring host defense peptides (HDPs), also named antimicrobial peptides, constitute an exciting class of drug candidates, especially because their mechanism of action presents less risk of inducing drug resistance. Indeed, the capacity of HDPs to interact with diverse cellular targets could explain that they have not yet generated widespread resistance^11,12^. HDPs are short cationic amphiphilic peptides that belong to the most ancient and conserved forms of innate immunity and exist across all major lineages. They display an unusually broad spectrum of activity against pathogens including bacteria, viruses, fungi and parasites^13^. Mammalian HDPs represent an important component of the innate immune system as they can trigger both direct microbe killing and rapid immune response modulation^14–17^.

Among all isolated and characterized HDPs, peptides generated from the endogenous processing of Chromogranin A are of particular therapeutic interest. Chromogranin A is an acidic protein stored in the secretory vesicles of numerous nervous, neuroendocrine and immune cells and is released upon stress in most of the body fluids including saliva^18–21^. Chromogranin A is known to be a precursor for several biological active peptides. Those peptides are linear, short (less than 25 residues) and therefore very easy to synthesize for a minimal cost. Moreover, they are stable in a wide range of temperature and pH ^22^.

Specifically, Catestatin (CGA_344–364_) has been reported to exhibit antimicrobial activity against a wide array of pathogens including bacteria, fungi and parasites^23–26^. Besides its crucial role as a catecholamine release inhibitor, Catestatin also triggers inflammation by exhibiting vasodilatation properties, activating neutrophils, attracting monocytes and mast cells, inducing mast cell degranulation and production of cytokines and chemokines^27–31^. Moreover, Catestatin is expressed in keratinocytes^32^. The arginine rich N-terminus fragment of Catestatin, named Cateslytin (Ctl; CGA_344–358_, RSMRLSFRARGYGFR) is an effective antimicrobial agent against several microbial strains including *C. albicans*^33,34^. Recently, we demonstrated that the dextrogyre (D) conformation of Ctl (D-Ctl) is much more potent than the natural peptide L-Ctl as an antibacterial agent^35^. Indeed, the substitution of some or all L-amino acids by D-amino acids increases the resistance of HDPs to proteolytic degradation^11,36^.

In the present study, we compared the activity of the two conformations of Ctl, levogyre (L) and dextrogyre (D), against *Candida albicans*. Our results, based on antifungal, safety and mechanistic assays reveal that D-Ctl shows the most effective antifungal properties towards *Candida albicans*, and could be used to treat its associated diseases including oral candidosis.

## Results

### Both D-Ctl and L-Ctl are potent antifungal agents against *Candida albicans*

**Figure 1:**
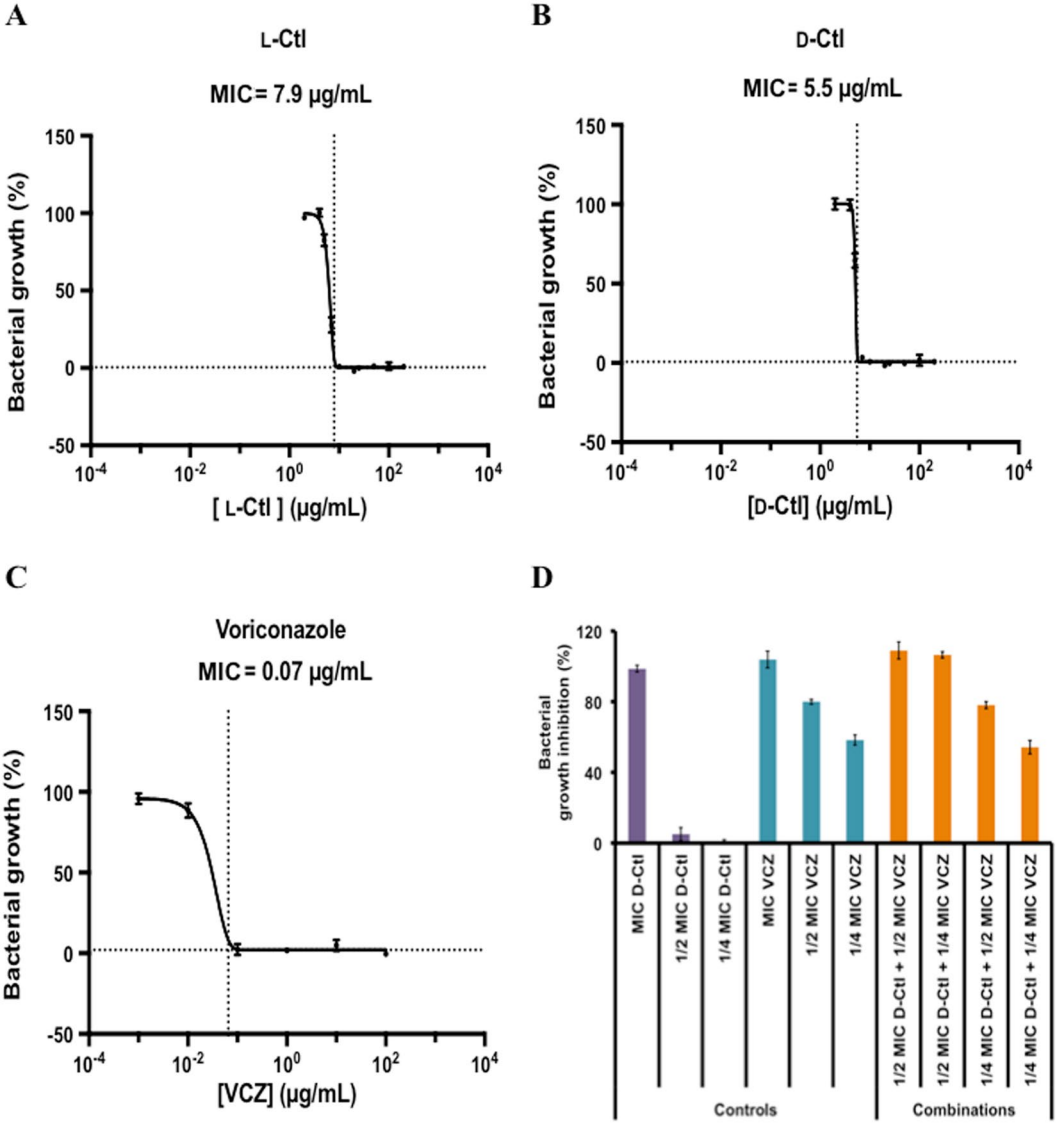
Antifungal activity of L-Ctl, D-Ctl, voriconazole (VCZ) and the combination of D-Ctl and VCZ against *Candida albicans*. Antifungal tests were performed in the presence of L-Ctl (**A**), D-Ctl (**B**), VCZ (**C**) or D-Ctl in combination with VCZ (**D**) as indicated. For each figure, the average of at least three separate experiments is shown. Each MIC, defined as the lowest concentration of antifungal agent (alone or in combination) able to inhibit 100% of *C. albicans* growth, was determined using a modified Gompertz model.

We first tested the potential of D-Ctl to inhibit *Candida albicans* growth using antifungal assays with different concentrations of D-Ctl, and compared it with L-Ctl. Our results show that D-Ctl displays a slightly better activity than L-Ctl with a minimal inhibitory concentration (MIC) of 5.5 μg/mL (2.9 μM) (Fig. 1B), compared to 7.9 μg/mL (4.2 μM) for L-Ctl (Fig. 1A).

### D-Ctl potentiates voriconazole, an antifungal of reference to treat *Candida albicans* associated infections

We then compared the activity of D-Ctl with voriconazole (VCZ), an antifungal of reference to treat *C. albicans* associated infections. For that purpose, antifungal assays were performed with increasing concentrations of VCZ. The MIC of VCZ was determined at 0.07 μg/mL (0.2 μM) (Fig. 1C).

Although really potent, VCZ-resistant *C. albicans* strains have been isolated^37^. One way to prevent the emergence of fungal resistance is to use combination therapy and further reduce the doses of VCZ prescribed. The effect of the combination between VCZ and D-Ctl was determined using antifungal assays combining different concentrations of both compounds (Fig. 1D). Our results show that the combination using the minimal amount of both antifungal agents and able to kill 100% of *Candida albicans* was ½ MIC_D-Ctl + _¼ MIC_VCZ_ with a FIC index of 0.75 (FIC index = FIC_VCZ_ + FIC_D-Ctl_ = 0.5 + 0.25 = 0.75). According to EUCAST^38^, a FIC index included between 0.5 and 1 indicates an additive antifungal effect of the combination. As a result, VCZ and D-Ctl have an additive effect on *C. albicans*. In other words, by adding D-Ctl to the treatment, the concentration of VCZ could be decreased by 4 (¼ MIC_VCZ =_ 0.018 μg/mL = 0.05 μM).

### D-Ctl and L-Ctl are not toxic for human gingival fibroblasts

**Figure 2:**
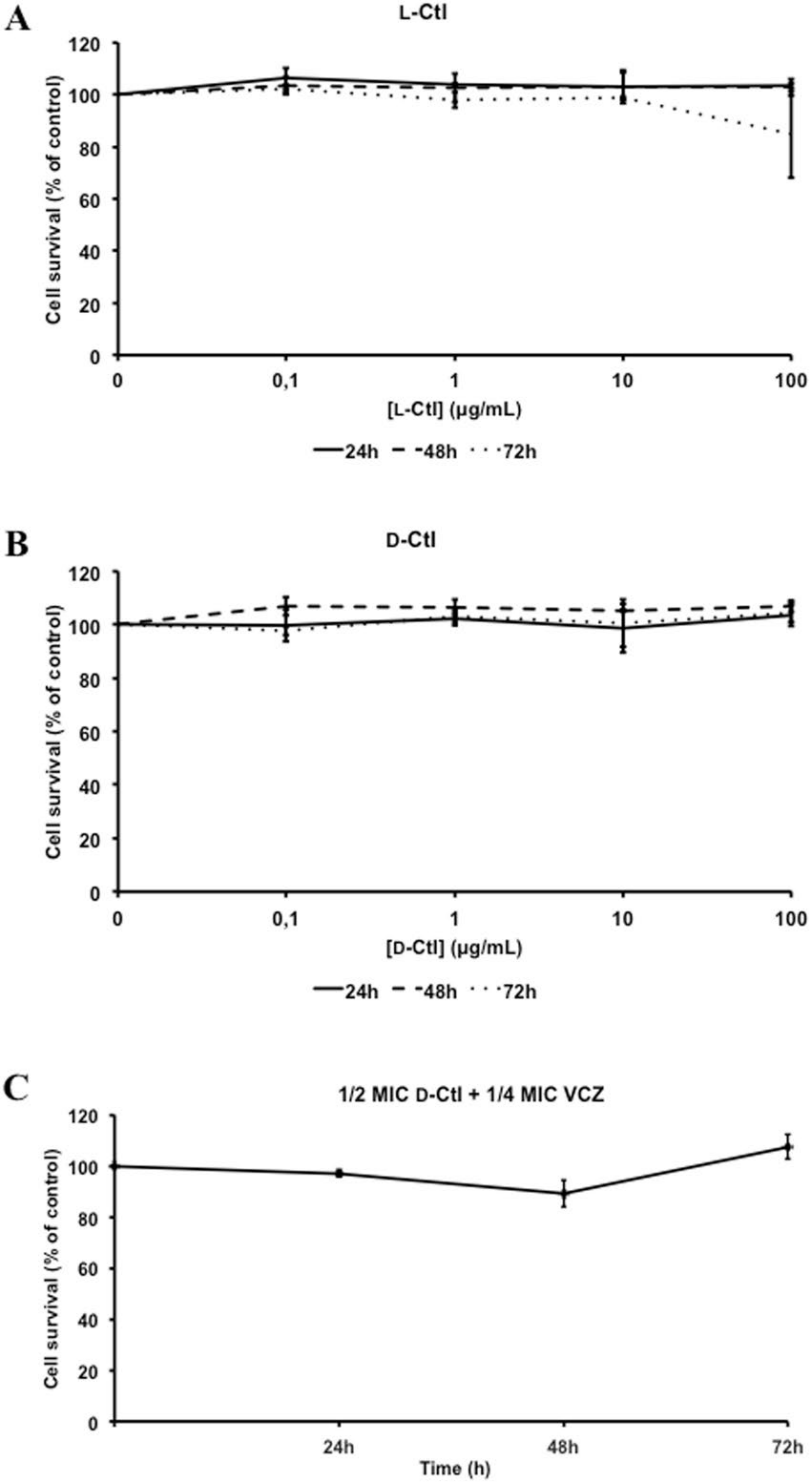
Cytotoxicity of L-Ctl and D-Ctl against human gingival fibroblasts (HGF-1) using MMT assays. L-Ctl (**A**) or D-Ctl (**B**) were incubated with HGF-1 cells at different concentrations (0 μg/mL, 0.1 μg/mL, 1 μg/mL, 10 μg/mL, 100 μg/mL) for 24 h, 48 h and 72 h. The combination ½ MIC D-Ctl + ¼ MIC VCZ (**C**) was also incubated with HGF-1 cells for 24 h, 48 h and 72 h. The results obtained are expressed as percentage of cell survival. For each panel, the average of at least three independent experiments is shown.

To assess the cytotoxicity of both peptides, we performed MTT assays using human gingival fibroblasts (HGF-1) as a cellular model. Thus, each peptide was incubated with the cells at different concentrations for 24 hours, 48 hours and 72 hours (Fig. 2). As expected for a host defense peptide, L-Ctl was not toxic at 100 μg/mL for a period of time ranging from 24 to 72 hours (Fig. 2A). Interestingly, D-Ctl was not cytotoxic either on HGF-1 after 72 hours for concentrations up to 100 μg/mL (Fig. 2B). As a result, neither L-Ctl nor D-Ctl showed cytotoxicity at their respective MIC. In addition, the combination of D-Ctl (½ MIC) and VCZ (¼ MIC) was also not toxic for human gingival fibroblasts (Fig. 2C).

### Unlike L-Ctl, D-Ctl is not degraded by the proteases secreted by *Candida albicans*

**Figure 3:**
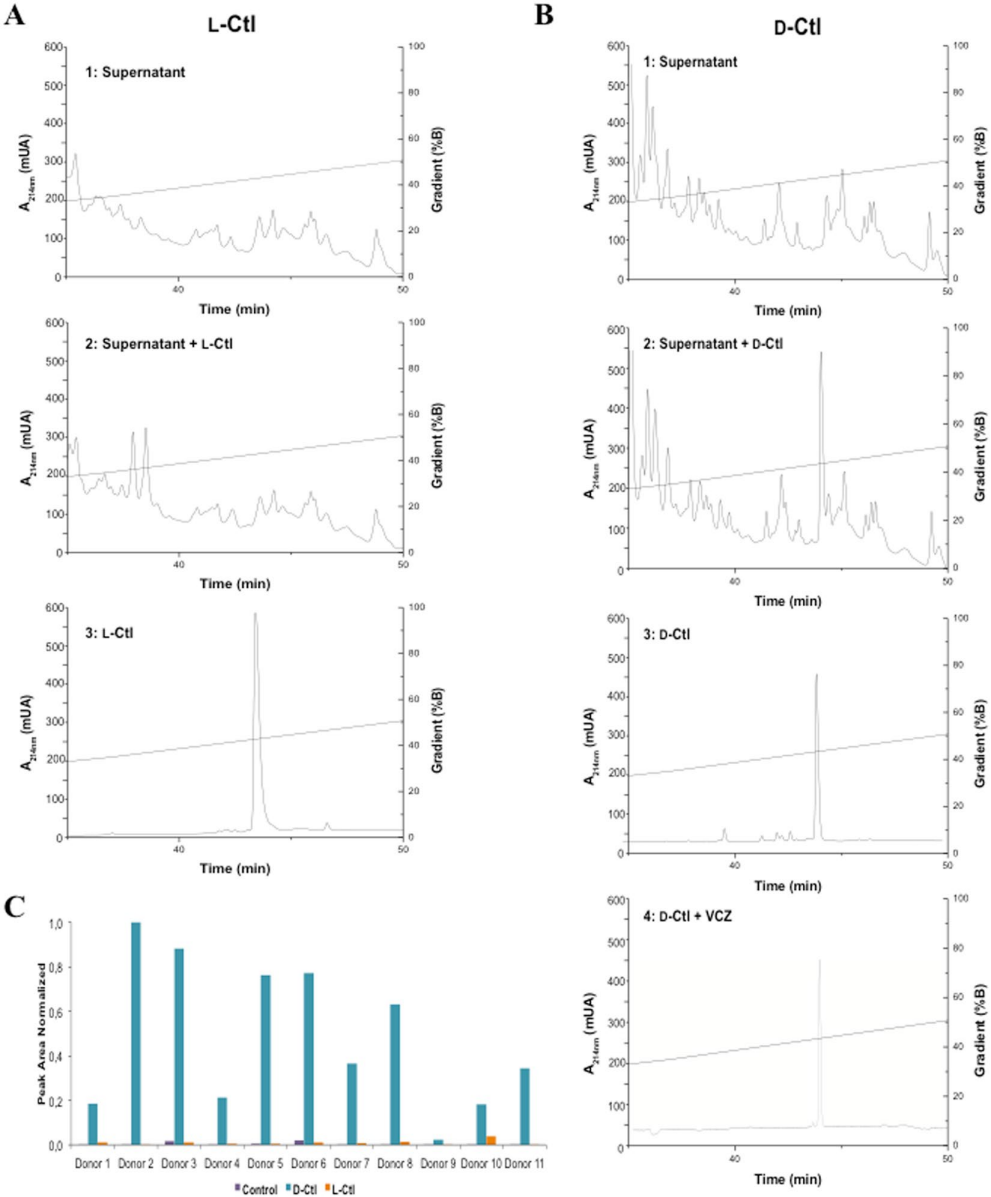
Stability of L-Ctl and D-Ctl in the supernatant of *Candida albicans* and in saliva. The supernatant of *Candida albicans* alone (chromatograms 1) or in the presence of the L-Ctl (**A**) or D-Ctl (**B**) (chromatograms 2) was directly incubated at 37 °C for 24 h prior being analysed by HPLC. As a control, each peptide (chromatograms 3) as well as the combination ½ MIC D-Ctl + ¼ MIC VCZ (chromatogram 4) were also incubated in water at 37 °C for 24 h. Each experiment was repeated three times. Saliva from 11 healthy volunteers was incubated with D-Ctl or L-Ctl for 24 hours and peptide stability was then assessed by LC-SRM (**C**).

In order to use D-Ctl as a therapeutic agent against *C. albicans*, it should not be degraded by its proteases. Subsequently, we tested whether D-Ctl was stable in the supernatant of *Candida albicans* by HPLC, compared to L-Ctl. To this aim, L-Ctl or D-Ctl were incubated in the supernatant of *Candida albicans* for 24 hours at 37 °C prior being analysed by HPLC (Fig. 3A,B). As a control, the peptides and the supernatant were incubated separately and analysed by HPLC.

Under our experimental conditions, both L-Ctl and D-Ctl had a retention time of 44 min (Fig. 3A,B, chromatograms 3). The profiles obtained for the supernatant of *Candida albicans* displayed numerous peaks corresponding to the peptides and proteins secreted by the pathogen (Fig. 3A,B, chromatograms 1). When L-Ctl was incubated with the supernatant of *Candida albicans*, the peak of L-Ctl disappeared from the chromatogram, suggesting that L-Ctl was degraded by proteases from the supernatant (Fig. 3A, chromatogram 2). However, D-Ctl was still present after an incubation of 24 hours, implying that it remains stable in the supernatant of *Candida albicans* (Fig. 3B, chromatogram 2). In addition, VCZ did not impact the stability of D-Ctl (Fig. 3B, chromatogram 4). These finding suggest that, as expected, the D-amino acids contribute to the stability of D-Ctl towards fungal proteases.

### Unlike L-Ctl, D-Ctl remains stable in saliva

Degradation during oral delivery is also a major concern for the use of an antifungal agent to treat oral candidosis. Consequently, we assessed the stability of D-Ctl compared to L-Ctl in the saliva of a cohort of eleven donors. Peptide integrity was measured after 24 hours incubation by LC-SRM (Fig. 3C). Remarkably, unlike L-Ctl, D-Ctl was stable in saliva for all donors tested. These results suggest that the D-conformation of Ctl can overcome the lack of stability of the natural peptide L-Ctl in saliva.

### D-Ctl quickly invades *Candida albicans* but only spreads from cell to cell during division

**Figure 4:**
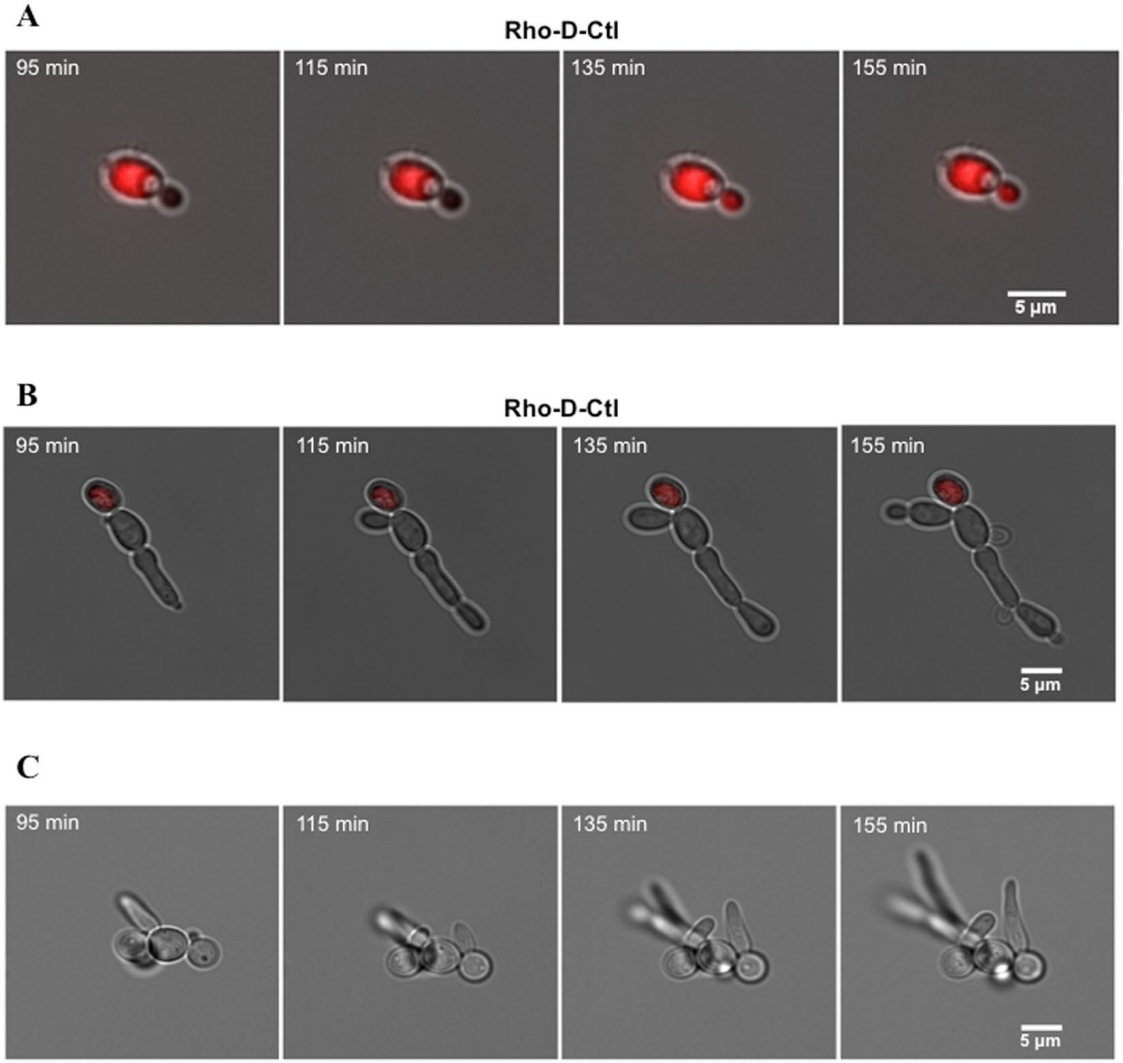
Time-lapse video microscopy of *Candida albicans* colonies treated with Rho-D-Ctl. *Candida albicans* was incubated for 30 min at 37 °C with Rho-D-Ctl at 10 × MIC (55 μg/mL). Cells were then washed and the interaction between *Candida albicans* and D-Ctl was followed by video microscopy. Images (fluorescence and phase contrast) were captured with a 60X objective. The time elapsed between two frames is 20 min.

To better understand the interaction between *C. albicans* and D-Ctl, we performed time-lapse imaging. Rhodamine-labelled D-Ctl and L-Ctl were incubated with *C. albicans* for 30 min. The excess of peptide was then removed to allow visualization of the peptide inside the yeast colonies. Both peptides were able to enter *C. albicans* during the 30 min incubation. Remarkably, after 95 min incubation with D-Ctl (Fig. 4A,B), we observed two groups of colonies: colonies totally invaded by D-Ctl (23/39 colonies observed = 59%) (Fig. 4A) and colonies partially invaded (16/39 colonies observed = 41%) (Fig. 4B). All colonies totally invaded by D-Ctl show a systematic arrest of fungal growth and ongoing cellular divisions (Fig. 4A). On the other hand, colonies partially invaded by D-Ctl could grow by division of the non-invaded cells (Fig. 4B). Interestingly, there was no observation of D-Ctl transiting from cell to cell unless during cellular division as shown in Fig. 4A (6/6 observed colonies = 100%). As a control, untreated *C. albicans* show normal growth over time (Fig. 4C). Altogether, these results demonstrate that D-Ctl can rapidly enter *C. albicans*, but is unable to spread within a yeast colony unless from a mother cell to a daughter cell during cellular division.

**Figure 5:**
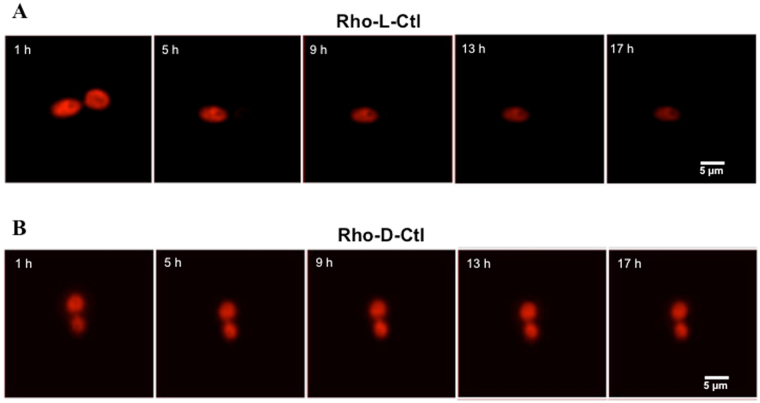
Comparison of the fluorescence intensity of *Candida albicans* treated with Rho-L-Ctl or Rho-D-Ctl. *Candida albicans* was incubated for 30 min at 37 °C with Rho-D-Ctl or Rho-L-Ctl at 10 × MIC (55 μg/mL and 79 μg/mL, respectively). Cells were then washed and the interaction between *C. albicans* and D-Ctl or L-Ctl was followed by video microscopy. Images were captured with a 60X objective. The time elapsed between two frames is 4 h.

Remarkably, over a period of 17 hours, we also observed that the intensity of fluorescence of rhodamine-labelled L-Ctl decreases (Fig. 5A) whereas it stays stable for rhodamine-labelled D-Ctl (Fig. 5B) (34/34 = 100% of L-Ctl treated colonies and 23/23 = 100% of D-Ctl treated colonies). This could be explained by the degradation of L-Ctl as previously described (Fig. 3A).

## Discussion

In the dental field, the most common pathology involving a fungal biofilm is oral candidosis. Oral candidosis manifests itself as an inflammatory process of the oral mucosa. *Candida albicans* is described as the main agent causing oral candidosis^4^. Oral candidosis is a public health issue that can affect patients at any age. Its prevalence is particularly strong in the increased risk populations such as elderly patients, diabetic, premature newborns or immunodepressed patients at risk of invasive fungal infections. The most important aspect of treatment is improving oral hygiene. A sialagogue may be useful for the recovery of a normal salivary flow in case of xerostomia, one of the symptoms occasionally associated with oral candidosis. The other aspect of treatment involves resolution of the mucosal infection, for which topical antifungal medications (as an oral suspension or a gel) are used. Systemic antifungal drugs are almost exclusively reserved for the patient with systemic factors that condition the development and persistence of oral candidosis, such as immunosuppression or diabetes. However, increased resistance to antifungal agents commonly prescribed motivates the search for new ways of fighting against oral candidosis^7–10^.

Naturally occurring host defense peptides (HDPs), also named antimicrobial peptides, constitute an exciting class of drugs candidates, especially because their mechanism of action presents less risk of inducing drug resistance^12^. In this context, we demonstrated here that D-Ctl, a derivative of L-Cateslytin (L-Ctl) is a potent antifungal against *C. albicans* and could be administered to treat oral candidosis as a monotherapy or in combination with voriconazole (VCZ), an antifungal agent of reference to treat *C. albicans* related infections. VCZ is effective for both mucosal and invasive candidosis, and specifically used to treat patients that are resistant, intolerant or presenting a contraindication to treatment with fluconazole or amphotericin B. The results from a six-year study (1996–2001) for 218 *Candida* species isolates causing bloodstream infection confirm the high efficiency of VCZ, described as the most active drug among all azole compounds tested (voriconazole, fluconazole, itraconazole) towards *C. albicans, C. parapsilosis, C. tropicalis* (80% of isolates), *C. krusei and C. glabrata*^39^. Actually, we demonstrated in this study that by adding D-Ctl to the treatment, the concentration of VCZ could be decreased by 4 with potential implications on the emergence of resistant phenotypes. One way to explain this synergistic effect would be that the peptide punches holes in the cell membrane, therefore facilitating the penetration of VCZ in the pathogen. Further investigation will be needed to better understand this mechanism.

When compared to VCZ, L-Ctl and D-Ctl were still less efficient against *C. albicans*. However, the powerful activity of most antifungal agents currently on the market is balanced by detrimental side effects. Indeed each triazole including fluconazole, itraconazole, voriconazole as well as amphotericin B has a profile of side effects ranging from rash, nausea, diarrhea, visual hallucinations to liver and/or kidney toxicity or heart failure^40^.

To validate the therapeutic potential of D-Ctl, we verified whether this peptide was not degraded by proteases secreted by *Candida albicans*. Indeed, as a mechanism of defence against the host, *C. albiacans* is able to release virulence factors such as the mannoprotein Mp65, the Seoul imipenemase Sim1 and the secreted aspartic protease Sap6^41^. As a result, unlike L-Ctl, D-Ctl was not degraded by the proteases secreted by *C. albicans*. Besides, in contrast to L-Ctl, D-Ctl was also stable in saliva (including two donors with gingivitis). Gingivitis manifests itself as an inflammation of the gingival tissue, which can lead to periodontitis. Actually these periodontal pathologies, as well as oral candidosis, induce qualitative and quantitative changes in the chemical composition of the saliva^42–44^. In this context, further experiments on saliva samples from donors affected by oral candidosis and/or periodontitis would be relevant to support the stability of D-Ctl. However the data obtained already suggest that D-Ctl could be used as a topical antifungal medication delivered in the oral cavity to treat oral candidosis.

In addition, time-lapse video microscopy reveals a quick invasion of D-Ctl within *Candida albicans*, without inducing cell lysis. Besides, D-Ctl was able to spread from a mother cell to a daughter cell only during cellular division to stop ongoing cellular divisions. In contrast with D-Ctl, the decreasing intensity of fluorescence of rhodamine-labelled L-Ctl over time suggests its degradation. One hypothesis could be that the resulting fragments of L-Ctl may then be released into the extracellular compartment, thus explaining the decreasing fluorescence in yeasts formations colonized by Rho-L-Ctl. Actually, little is known about the mechanisms by which cationic HDPs enter or escape cells. However, recent experiments indicate that Ctl, as well as closely related cationic peptides, develop an α-helix-β-sheet structure when in contact with negatively charged membrane interfaces, resulting in phospholipid membrane deformations and pore formations^45,46^. In addition, further investigations will be needed to clearly identify the target of D-Ctl within the cells of *Candida albicans*.

Altogether, our study suggests that D-Ctl constitutes an excellent candidate for the development of a new antifungal agent against *Candida albicans*. The antifungal protection of the mucosa is an indication of the first order, as a possible prevention of oral fungal infections. A topical application (as a suspension or a gel) of such an antifungal agent therefore constitutes an interesting pathway for the protection of oral mucosa. Further investigations will thus be needed to develop D-Ctl-based gels or suspensions for topical applications in the treatment of oral candidosis. From this perspective, it would be relevant to complement the data obtained with the evaluation of the cytotoxicity of D-Ctl towards epithelial cells of the oral mucosa.

## Methods

### Peptide synthesis

L-Ctl (CGA_344–358_, RSMRLSFRARGYGFR) and its derivate D-Ctl, as well as the rhodamined peptides Rho-L-Ctl and Rho-D-Ctl, were synthesized by Proteogenix SAS according to the Merrifield Technique, a stepwise solid-phase peptide synthesis approach with FMOC chemistry, and purified to >95% by MALDI-TOF mass spectrometry and reverse phase high-performance liquid chromatography (RP-HPLC). The rhodamine moiety added to L-Ctl and D-Ctl and used for video microscopy is located on the N-terminal end of the polypeptidic chain.

### Antifungal tests

*Candida albicans* (ATCC© 10231^TM^) was cultured in Sabouraud medium (Sigma-Aldrich) supplemented with tetracycline (10 μg/mL) and cefotaxime (10 μg/mL) at 37 °C for 24 h. *Candida albicans* (OD_600nm_ = 0,001) were plated in 96-well plates and treated either with different concentrations of the peptides of interest, and/or with different concentrations of voriconazole (VCZ) (Sigma-Aldrich). As a positive control, cells were treated with 10 μg/mL VCZ. After 24 hours incubation, yeast growth was assessed by optical density OD_600nm_ using a spectrophotometer (Multiscan EX).

The MIC of the antifungal agents, defined as the lowest concentration of drug able to inhibit 100% of the growth of a pathogen was determined using a modified Gompertz model ^47^.

The efficiency of the combination between D-Ctl and VCZ was determined by the calculation of the Fractional inhibitory concentration index (FICI) according to the following formula: FICI = FIC_D-Ctl_ + FIC_VCZ_ = (MIC_D-Ctl_ in combination/MIC_D-Ctl_ ${\rm{alone}})$ + (MIC_VCZ_ in combination/MIC_VCZ_ ${\rm{alone}})$. According to EUCAST^38^, the effect of the combination is synergistic if FICI ≤ 0,5, additive if 0,5 < FICI ≤ 1, indifferent if 1 < FICI < 2 and antagonistic if FICI ≥ 2.

### Cell viability assays

Peptide cytotoxicity was assessed by MMT [3-(4,5-dimethylthiazol-2-yl)-2,5 diphenyl tetrazolium bromide] assays (Sigma-Aldrich) using a human gingival fibroblasts (HGF-1) cell line (ATCC® CRL-2014^TM^) as a model. HGF-1 cells were maintained in DMEM (Sigma-Aldrich) supplemented with 10% fetal bovine serum (Gibco) and 1% penicillin/streptomycin (Sigma-Aldrich) at 37 °C and 5% CO_2_.

HGF-1 cells (10^6^ cellules/mL) were plated in 96-well plates for 24 hours, prior being treated with different concentrations of L-Ctl, D-Ctl or the combination ½ MIC_D-Ctl_  + ¼ MIC_VCZ_ for 24, 48 or 72 hours. The culture media was then removed and replaced by MTT diluted in culture media (0,25 mg/mL). Cells were then incubated for an additional 3 hours at 37 °C, 5% CO_2_ and lysed with isopropanol/HCl (v/v). After 15 min incubation at room temperature and under agitation, cell viability was assessed by optical density OD_550nm_ using a spectrophotometer (Multiscan EX).

### Peptide stability assays in the supernatant of Candida albicans

*The supernatant of Candida albicans* was prepared as follows: a single colony of *Candida albicans* was resuspended in 5 mL of Sabouraud medium and incubated at 37 °C overnight. The culture was then centrifuged at 10000 g for 1 min and the supernatant was filtered using a 0.22 μm MillexH-GV (Merck Millipore). In order to check sterility, an aliquot of the supernatant was incubated at 37 °C for 48 h. The absence of growth was interpreted as a lack of viable microorganism.

100 μL of supernatant was then directly incubated or not with L-Ctl or D-Ctl (186 μg/mL = 100 μM) at 37 °C for 24 h. As a control, each peptide (186 μg/mL = 100 μM) as well as the combination ½ MIC_D-Ctl_ + ¼ MIC_VCZ_ were incubated in water (100 μL) at 37 °C for 24 h. Samples were then separated using a Dionex HPLC system (Ultimate 3000) on a Nucleosil reverse-phase 300–5C18-column (4.6 × 250 mm; particle size: 5 μm; porosity, 300 Å) (Macherey Nagel). Absorbance was monitored at 214 nm and the solvent system consisted of 0.1% (v/v) TFA in water (solvent A) and 0.09% (v/v) TFA in 70% (v/v) acetonitrile-water (solvent B). Elution was performed at a flow rate of 700 μL/min with a gradient of solvent B as indicated on the chromatograms. Each peak was manually collected.

### Peptide stability assays in saliva by LC-SRM

Saliva samples were obtained from a cohort of 11 donors (4 men and 7 women) and collected at the Faculty of Dentistry of the University of Strasbourg (France) (see Supplemental Material for donors characteristics). According to European and French legislation, institutional review board or ethics committee approval was not required for this study. Informed consent was obtained from all donors.

Samples were prepared as follows: 100 μL of saliva was directly incubated with or without each peptide of interest (230 μg/mL = 123 μM) at 37 °C for 24 h. As a control, each peptide (230 μg/mL = 123 μM) was incubated in water (100 μL) at 37 °C for 24 h. Samples were then centrifuged at 14 000 g during 5 min at 4 °C and then diluted 3 times in water with 0.1% (v/v) HCOOH. For each sample, 2 µL were injected on the LC-SRM system. All separations were carried out on an Agilent 1100 Series HPLC system (Agilent Technologies). For each analysis, the sample was loaded into a trapping column ZORBAX 300SB-C18 MicroBore Guard 5 µm, 1.0 × 17 mm (Agilent Technologies) at 50 µL/min with aqueous solution containing 0.1% (v/v) HCOOH and 2% CH3CN. After 3 min trapping, the column was put on-line with a ZORBAX 300SB-C18 3.5 µm, 0.3 × 150 mm column (Agilent Technologies). Peptide elution was performed at 5 µL/min by applying a linear gradient of solvent A (water with 0.1% (v/v) HCOOH) and B (CH3CN with 0.1% (v/v) HCOOH), from 40 to 95% solvent B over 5 min followed by a washing step (3 min at 95% solvent B) and an equilibration step (13 min at 40% solvent B).

For LC-SRM, the antimicrobial peptide was targeted with either an oxidized or non-oxidized state [RSMRLSFRARGYGFR]. For each state, three different precursors corresponding to three different charged states (3+, 4+ and 5+) were followed. For each of the 6 precursors, 8 transitions were monitored (48 transitions in total) on a QQQ-6490 triple quadrupole mass spectrometer (Agilent technologies) in unscheduled mode and within a cycle time of 3 000 ms. For each transition, the collision energy was experimentally optimized by testing 7 values (step of ±3 V) centred on the reference value. The reference value was calculated using the equation given by the supplier. The isolation width for both Q1 and Q3 was set to 0.7 m/z unit. Mass data collected during LC-SRM were processed with the Skyline open-source software package 3.6.1^48^. Area intensities of each precursor were manually checked.

### Time-lapse video microscopy

Diluted precultures (1/1000) of *Candida albicans* were incubated for 1 hour at 37 °C without agitation in glass bottom μ-Dish^35mm,high^ (Ibidi) previously treated with poly-L-lysine. The rhodamine-labelled peptides (Rho-L-Ctl or Rho-D-Ctl) were then added to the culture at a concentration of 10X MIC and incubated for 30 min at 37 °C under agitation. To perform time-lapse video microscopy, the peptides were removed and replaced by Sabouraud medium. Yeasts were then plated at 37 °C, 5% CO_2_, on a Nikon Eclipse Ti inverted microscope equipped with an Andor Zyla sCMOS camera and a 60X objective. To prevent evaporation, a layer of mineral oil (Sigma) was added on top of the media prior imaging. Images (fluorescence and phase contrast) were captured every 10 min for 20 hours using Nikon NIS-Elements AR software, and processed with ImageJ.

## Electronic supplementary material

Supplementary Dataset 1
