# Supplementary material for: D-Cateslytin: a new antifungal agent for the treatment of oral Candida albicans associated infections
Source: Sci Rep. 2018 Jun 18;8:9235. doi: 10.1038/s41598-018-27417-x (PMC6006364; doi:10.1038/s41598-018-27417-x)
Supplement: Supplementary file 1 — Supplementary Dataset 1 [file 41598_2018_27417_MOESM1_ESM.doc]

**D-Cateslytin: a new antifungal agent for the treatment of oral *Candida albicans* associated infections**

**Pauline Dartevelle^1, 2, 3^, Claire Ehlinger^1, 2, 3^, Abdurraouf Zaet^1, 2, 3^, Christian Boehler^1, 2, 3^, Morgane Rabineau^1, 3^, Benoit Westermann^4^, Jean-Marc Strub^4^, Sarah Cianferani^4^, Youssef Haïkel^1, 2, 3^, Marie-Hélène Metz-Boutigue^1, 3^, Céline Marban^1, 2, 3*^**

^1^ INSERM UMR 1121, Biomatériaux et Bioingénierie, Université de Strasbourg, 11 rue Humann 67085 Strasbourg France

^2^ Faculté de Chirurgie Dentaire, Université de Strasbourg, 3 rue Sainte Elisabeth 67000 Strasbourg France

^3^ Fédération de Médecine Translationnelle, Université de Strasbourg, France

^4^ Laboratoire de spectrométrie de masse bioorganique, Université de Strasbourg, CNRS UMR 7178 Strasbourg, France

^*^ Corresponding author: celinemarban@gmail.com

**SUPPLEMENTAL FIGURE LEGENDS**


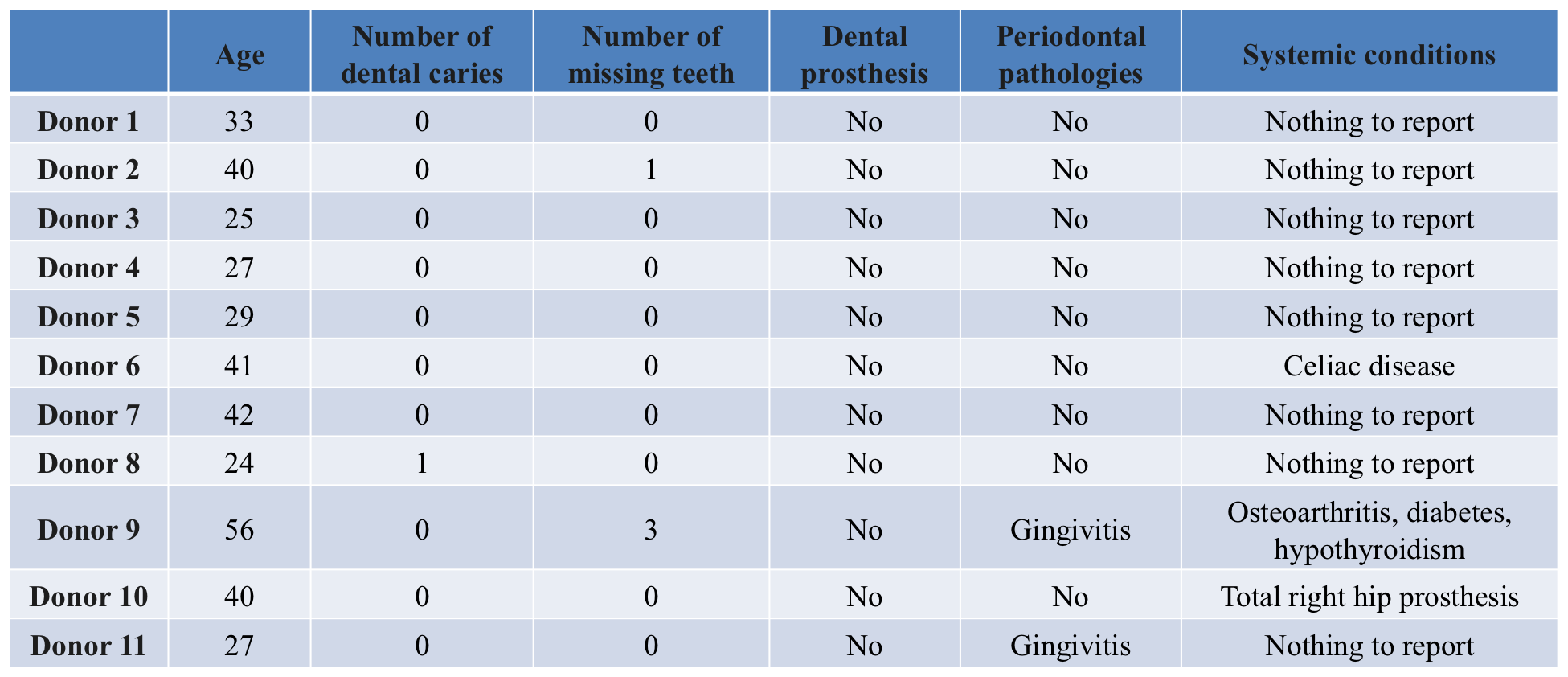
**Supplementary Table S1: Characteristics of the cohort.** Oral patients situation including the presence and/or absence of dental caries, number of lost teeth, presence of prosthesis and periodontal aspects, age and systemic conditions is indicated.
